# Breast Axillary Lymph Node Metastasis

**DOI:** 10.4061/2011/304697

**Published:** 2012-02-13

**Authors:** Luciane R. Cavalli, Rachel E. Ellsworth, Christoph Klein, Giuseppe Viale

**Affiliations:** ^1^Department of Oncology, Lombardi Comprehensive Cancer Center, Georgetown University, 3800 Reservoir Road NW, Washington, DC 20007, USA; ^2^Clinical Breast Care Project, 620 Seventh Street, Windber, PA 15963, USA; ^3^Division of Oncogenomics, Department of Pathology, University of Regensburg, Franz-Josef-Strauß-Allee 11, 93053 Regensburg, Germany; ^4^Department of Pathology, University of Milan School of Medicine and European Institute of Oncology, Via Ripamonti 435, 20141 Milan, Italy

The surgical management of breast cancer has evolved over the years from extensive radical mastectomy to breast conservation surgery. Until the introduction of the sentinel lymph node biopsy (SLNB), all patients with invasive breast cancer would undergo complete axillary lymph node dissection (ALND) and thus be at risk of suffering from its associated high morbidity. SLNB has become the standard of care and represented significant progress toward reducing the invasive procedures for the management of the axilla. The most recent clinical trials (NSABP-B32 and ACOSOG Z0011) performed in patients that underwent SLNB support this procedure as an accurate predictor of the risk of further axillary node involvement and of breast cancer recurrence. Additionally, the ACOSOG Z0011 trial challenged the standard of practice in the management of the axilla, in which ALND is mandated for all the patients with a positive sentinel node. A similar outcome was demonstrated for a selected group of patients (treated with breast conserving surgery and radiotherapy) with positive SLNB then followed by ALND or SLNB only. In light of these results, the role of ALND in the current management of breast cancer is being reevaluated for specific patient subpopulations. In this special issue, this timely subject is provocatively reviewed in addition to other relevant topics, such as the controversial meaning of the presence of micrometastasis and isolated tumor cells in the SLN in relation to local recurrence and overall survival and the feasibility of performing SLNB after neoadjuvant treatment and transaxillary breast augmentation.

Despite the advances in the lymphatic mapping and in the intraoperative methods for SLN analysis, the accurate identification of tumor cells in this node continues to be a challenge in clinical practice, and significant false-negative results in SLNB are still observed. In a research study published in this issue, a preferential cellular distribution of the malignant cells in the SLN is reported, suggesting that the pathological analysis directed to this area may contribute to a more precise identification of nodal metastasis. Additional progress in this direction involves the development of molecular markers, which would tackle not only the misdiagnosed SLN-negative patients but also the ones with low risk of recurrence that are unnecessarily submitted to SLNB. In this sense, the Bayesian-based nomogram developed by Westover et al. is proposed to be particularly useful, especially in cases where the SLNB assessment is predicted to be less sensitive. This nomogram would also lead to the identification of high-risk individuals for recurrence, based on the calculation of residual axillary disease risk, despite a negative SLNB. 

Several new reports, mostly based on gene expression profiling, have suggested that the different rates of recurrence can be due to the distinct molecular types of breast cancer. The development of a genomic signature that effectively discriminates patients by lymph node status, as the one proposed by Ellsworth et al., could stratify patients based on their need of surgical evaluation of the lymph nodes, sparing the ones in which disease will probably be limited to its primary site. The assessment of specific tumor markers in the lymph nodes is also discussed in this special issue, including a review of the prognostic and/or predictive implications of lymph node metastasis in tumors with elevated levels of CXCR4 (a protein chemokine receptor) and VEGF-C (a vascular endothelial cellular growth factor).

From the Halsted radical mastectomy to the commercial gene expression profiling tests, axillary lymph node management and recurrence prediction are still evolving topics for patients with breast cancer. The continued improvement of molecular tumor profiling and bioinformatics from larger and better defined patient cohorts will certainly provide answers to many challenging questions regarding the axillary metastatic process. The clarification of this complex molecular mechanism and the identification of novel and integrative molecular markers that can reliably predict lymph node involvement that will affect risk of recurrence and survival will continue to form the basis of the contemporary approach for breast cancer management, where an early prediction of axillary metastasis and a personalized cancer treatment can be achieved.

*Luciane R. Cavalli* *Luciane R. Cavalli* *Rachel E. Ellsworth* *Rachel E. Ellsworth* *Christoph Klein* *Christoph Klein* *Giuseppe Viale* *Giuseppe Viale*

